# *Chlamydia trachomatis* Inhibits Homologous Recombination Repair of DNA Breaks by Interfering with PP2A Signaling

**DOI:** 10.1128/mBio.01465-18

**Published:** 2018-11-06

**Authors:** Yang Mi, Rajendra Kumar Gurumurthy, Piotr K. Zadora, Thomas F. Meyer, Cindrilla Chumduri

**Affiliations:** a Department of Molecular Biology, Max Planck Institute for Infection Biology, Berlin, Germany; b Key Laboratory of H. pylori and Gastrointestinal Microecology of Henan Province, The Fifth Affiliated Hospital of Zhengzhou University, Zhengzhou, Henan, China; c Department of Hepatology and Gastroenterology, Charité University Medicine, Berlin, Germany; University of Washington

**Keywords:** DNA damage response, DNA double-strand breaks, ATM, PP2A, homologous recombination repair, infection

## Abstract

Cervical and ovarian cancers exhibit characteristic mutational signatures that are reminiscent of mutational processes, including defective homologous recombination (HR) repair. How these mutational processes are initiated during carcinogenesis is largely unclear. Chlamydia trachomatis infections are epidemiologically associated with cervical and ovarian cancers. Previously, we showed that C. trachomatis induces DNA double-strand breaks (DSBs) but suppresses Ataxia-telangiectasia mutated (ATM) activation and cell cycle checkpoints. The mechanisms by which ATM regulation is modulated and its consequences for the repair pathway in C. trachomatis-infected cells remain unknown. Here, we found that *Chlamydia* bacteria interfere with the usual response of PP2A to DSBs. As a result, PP2A activity remains high, as the level of inhibitory phosphorylation at Y307 remains unchanged following C. trachomatis-induced DSBs. Protein-protein interaction analysis revealed that C. trachomatis facilitates persistent interactions of PP2A with ATM, thus suppressing ATM activation. This correlated with a remarkable lack of homologous recombination (HR) repair in C. trachomatis-infected cells. Chemical inhibition of PP2A activity in infected cells released ATM from PP2A, resulting in ATM phosphorylation. Activated ATM was then recruited to DSBs and initiated downstream signaling, including phosphorylation of MRE11 and NBS1 and checkpoint kinase 2 (Chk2)-mediated activation of the G_2_/M cell cycle checkpoint in C. trachomatis-infected cells. Further, PP2A inhibition led to the restoration of C. trachomatis-suppressed HR DNA repair function. Taking the data together, this study revealed that C. trachomatis modulates PP2A signaling to suppress ATM activation to prevent cell cycle arrest, thus contributing to a deficient high-fidelity HR pathway and a conducive environment for mutagenesis.

## Abstract (precis)

Chlamydia trachomatis induces DNA double-strand breaks in host cells but simultaneously inhibits proper DNA damage response and repair mechanisms. This may render host cells prone to loss of genetic integrity and transformation. Here we show that C. trachomatis prevents activation of the key DNA damage response mediator ATM by preventing the release from PP2A, leading to a complete absence of homologous recombination repair in host cells.

## INTRODUCTION

In recent years, several epidemiological studies have implicated infections with the Gram-negative bacterial pathogen Chlamydia trachomatis in the development of cervical (1, 2) and ovarian (3) carcinomas. These cancers together show eight distinct validated mutational signatures (http://cancer.sanger.ac.uk/cosmic/signatures), one of which is attributed to defective homologous recombination (HR) repair. How these mutational processes are initiated during the course of carcinogenesis is largely unknown. It is thus intriguing that C. trachomatis, an obligate intracellular bacterium causing chronic, asymptomatic infections, promotes DNA double-strand breaks (DSBs) and modulates a range of host cellular functions and signal transduction pathways, including those involved in preserving cellular and genomic integrity (4–10) and immune activation and apoptosis induction (11).

DSBs represent the most dangerous form of damage, as they cannot always be correctly repaired and thus pose the risk of genomic instability and chromosomal rearrangements (12). The DNA damage response (DDR) in mammalian cells prevents the accumulation of mutations and genomic instability (12, 13). Ataxia-telangiectasia mutated (ATM), an apical activator induced by DSBs, plays a critical role in relaying a strong, widespread signal to numerous downstream effectors involved in multiple processes (14), including repair, cell cycle, and cell death (15). Activation of ATM can occur by direct interaction with single-stranded DNA or oligonucleotides at DSBs, as well as by a direct interaction with the MRE11-RAD50-NBS1 (MRN) complex, whose members are themselves ATM substrates (16). Thus, a positive-feedback loop between ATM and its substrate proteins seems to be required for its correct positioning at break sites.

In our previous study, we showed that *Chlamydia* induces DSBs but simultaneously suppresses the activation and recruitment of ATM and MRE11 to the damage sites (8, 17). However, the molecular mechanisms by which *Chlamydia* suppresses activation of ATM signaling in the face of extensive DSBs and its consequences for the function of the error-free HR repair pathway remain unknown. Phosphorylation and dephosphorylation of proteins appear to be crucial for activating the DDR within minutes of DNA damage (18), suggesting a prime role for protein phosphatases in regulating the DDR (16). Protein phosphatase 2A (PP2A), a serine/threonine phosphatase, has been implicated in regulation of ATM activity in response to radiation-induced DSBs (19). PP2A holoenzymes are heterotrimers consisting of a core dimer scaffold (A) and a catalytic (C) subunit that is associated with one of the regulatory (B) subunits. Posttranslational modification in the C-terminal part of the catalytic subunit regulates the phosphatase activity of PP2A. Phosphorylation of tyrosine residue 307 (Y307) on the C subunit results in decreased PP2A enzyme activity (20, 21). Upon the formation of irradiation-induced DSBs, the PP2AC-B55α regulatory subunit of PP2A, which normally facilitates association with ATM, rapidly dissociates, leading to ATM autophosphorylation and activation (19, 22).

Here, we addressed the involvement of PP2A in the failure to mount an adequate response to DSBs in *Chlamydia*-infected cells. Interestingly, PP2A activity remained high and exhibited a persistent interaction with ATM, keeping it in an inactive state despite the DSBs. This is in line with data indicating a remarkable loss of HR repair function in C. trachomatis-infected cells. Treatment with okadaic acid (OA), an inhibitor of PP2A, led to increased phosphorylation of Y307 on the PP2A catalytic subunit and released ATM from PP2A, resulting in ATM phosphorylation. The activated ATM was then recruited to chromatin and initiated downstream signaling, leading to checkpoint kinase 2 (Chk2)-mediated G_2_/M cell cycle checkpoint activation and restoration of HR DNA repair. Taking the data together, this study revealed an intriguing mechanism by which *Chlamydia* modulates host signaling to support its intracellular development. By inhibiting ATM signaling, this pathogen inactivates an essential high-fidelity HR pathway and predisposes infected cells to mutagenesis.

## RESULTS

### Chlamydia trachomatis-induced ATM inactivation is mediated by protein phosphatase 2A.

**FIG 1:**
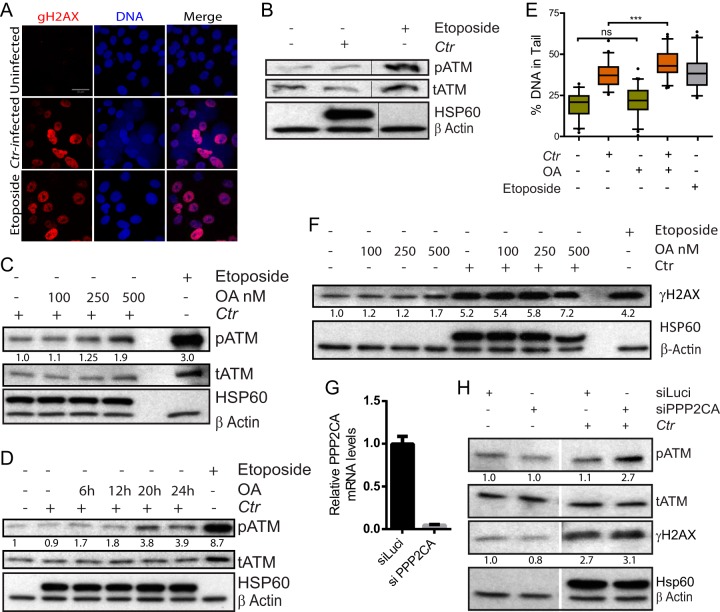
*Chlamydia*-induced DNA double-strand breaks fail to activate ATM by engaging PP2A. (A to F) Human cervical epithelial End1 E6/E7 cells were infected with C. trachomatis (*Ctr*) and (A) labeled for γH2AX and DNA (Draq5) at 36 h and (B) analyzed by immunoblotting for pATM, total ATM (tATM), chlamydial Hsp60, and β-actin at 45 h postinfection (h p.i.). (C and D) Cells were analyzed as described for panel B following treatment with OA (C) at different concentrations from 2 h before harvesting or (D) at 20 nM for different periods. (E) Cells were treated with OA (250 nM) from 2 h before harvesting at 45 h p.i. followed by neutral comet assay to assess quantity of DSBs. (F) Cells were treated with OA at the concentrations indicated from 2 h before harvesting at 45 h p.i. followed by immunoblotting for γH2AX, chlamydial Hsp60, and β-actin. (A to F) Cells treated with 50 µM etoposide for 2 h before analysis served as a positive control. (G and H) END1 E6/E7 cells transfected with siRNAs targeting luciferase or PPP2CA were harvested 45 h after infection with C. trachomatis and analyzed for (G) knockdown efficiency by quantitative reverse transcription-PCR (qRT-PCR) and (H) pATM, tATM, γH2AX, chlamydial Hsp60, and β-actin levels by immunoblotting. Data represent means ± standard deviations (SD) of results from three experiments normalized to mock-treated infected cells. Representative blots of three independent experiments are shown; Densitometry values for pATM and γH2AX immunoblots were normalized to the β-actin values, and data representing the relative fold change compared to control are shown.***, *P* < 0.001; *, *P* < 0.05; ns, *P* > 0.05 (determined by Student's *t* test).

Irradiation-induced DSBs elicit PP2A-mediated ATM activation (18). However, C. trachomatis infection suppresses the phosphorylation-mediated activation of ATM despite induction of extensive DSBs (Fig. 1A and B). Here we investigated the role of PP2A in regulating ATM suppression after the formation of C. trachomatis*-*induced DSBs. Treatment of C. trachomatis-infected cells with the protein phosphatase inhibitor okadaic acid (OA) at different concentrations (19) for the last 2 h or for different periods of time during infection led to increased phosphorylation of ATM at Ser1981 (pATM) in a concentration- and exposure time-dependent manner compared to untreated infected cells (Fig. 1C and D). While OA treatment itself did not induce DSBs, OA treatment of C. trachomatis-infected cells led to enhanced induction of DSBs, as demonstrated by neutral comet assay (Fig. 1E). However, no increase in the levels of phospho-H2AX (S139) (γH2AX), a hallmark of DSBs, was observed as demonstrated by immunoblot analysis (Fig. 1F). Further, to analyze the specific role of PP2A in the ATM inhibition observed in C. trachomatis-infected cells, small interfering RNA (siRNA)-mediated knockdown (KD) of the PP2A catalytic alpha subunit PPP2CA was performed. PPP2CA KD led to ATM activation in C. trachomatis-infected cells (Fig. 1G and H) in contrast to C. trachomatis-infected cells transfected with a neutral control (siLuci). Thus, PP2A plays a key role in the suppression of ATM activation after C. trachomatis induction of DSBs.

### Dynamic interaction of ATM and PP2A persists despite the presence of *Chlamydia*-induced DNA double-strand breaks.

**FIG 2:**
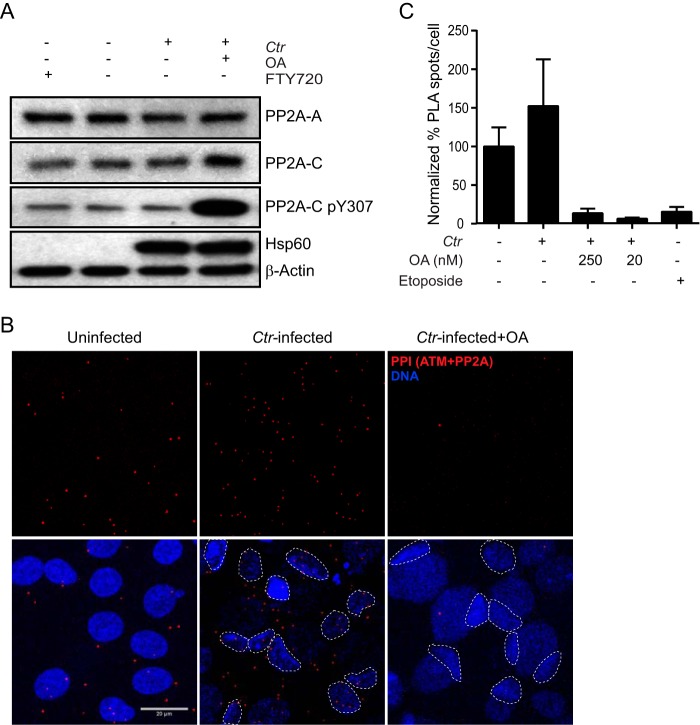
Inhibition of PP2A disrupts the dynamic interaction between PP2A and ATM in C. trachomatis-infected cells. (A) End1 E6/E7 cells infected with C. trachomatis with or without treatment with OA for the last 20 h were subjected to immunoblotting for PP2A-A, PP2A-C, PP2A-C pY307, chlamydial Hsp60, and β-actin at 45 h p.i. Cells treated with the chemical compound FTY72, which increases PP2A activity, were used as a positive control. (B and C) Uninfected and C. trachomatis-infected End1 E6/E7 cells were labeled by the use of a Duolink *in situ* PLA kit with antibodies against ATM and PP2A. (B) Fluorescent dots represent interactions between ATM and PP2A. Host nuclei are marked with dotted lines. Images shown are representative of results from three independent experiments. Bar, 20 μm. (C) Number of ATM and PP2A interactions normalized to control uninfected untreated cells, shown as means + standard errors of the means (SEM). Blot represents results of three independent experiments.

Phosphorylation of PP2A catalytic subunit C at Y307 (PP2A-C pY307) inhibits binding of the regulatory subunit B, an essential component for interaction with target proteins. To evaluate whether functionally active PP2A is directly responsible for inhibiting ATM activation, we analyzed the level of PP2A-C pY307 in C. trachomatis-infected cells with and without OA treatment. The levels of pY307 remained unaffected in response to C. trachomatis infection or treatment with FTY720, an immunomodulator that activates PP2A, which served as a positive control (Fig. 2A). Interestingly, a dramatic increase in PP2A-C pY307 levels in C. trachomatis-infected cells treated with OA was observed, indicating that PP2A was present in its active form in the C. trachomatis-infected cells. Further, we performed an *in situ* proximity ligation assay (PLA) using specific antibodies against PP2A and ATM to visualize protein-protein interactions. We observed a stronger interaction between PP2A and ATM in C. trachomatis-infected cells than in uninfected cells. However, additional treatment of C. trachomatis-infected cells with OA led to reduced interactions of PP2A and ATM, similarly to what is observed after etoposide-induced DSBs (Fig. 2B and C). Taking the data together, PP2A remains active in C. trachomatis*-*infected cells and continues to interact with ATM, thus maintaining it in a dephosphorylated state despite the presence of DSBs.

### The G_2_/M cell cycle checkpoint is activated in C. trachomatis-infected cells upon PP2A inhibition.

**FIG 3:**
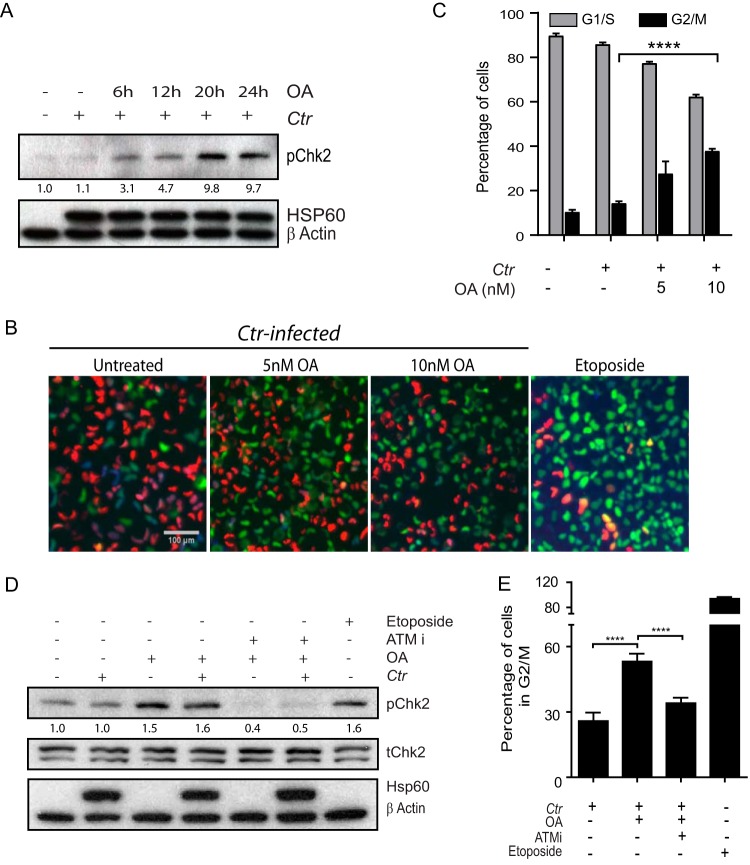
ATM activation upon PP2A inhibition induces cell cycle checkpoint activation and G_2_/M arrest in C. trachomatis-infected cells. (A) C. trachomatis-infected End1 E6/E7 cells with or without OA added at the indicated time points before harvesting at 45 h p.i. were analyzed for pChk2, chlamydial Hsp60, and β-actin by immunoblotting. (B and C) C. trachomatis-infected HeLa FUCCI cells were treated with OA for 20 h before fixing was performed at 45 h p.i. Cells were pulsed with BrdU for 1 h before immunofluorescent labeling for BrdU (blue), and DNA was counterstained with Hoechst stain. (B) Representative images of cells in G_1_ phase (red), G_2_/M phase (green), and S phase (positive for red and blue [BrdU] signal). Bar, 100 μm. (C) Percentages of cells in G_1_/S phase (red positive [red^+^]/BrdU^+^) and G_2_/M phase (green^+^/BrdU^−^). (D) C. trachomatis-infected End1 E6/E7 cells were treated with OA and ATMi 2 h before harvesting at 45 h p.i. followed by immunoblot analysis of pChk2, tChk2, chlamydial Hsp60, and β-actin. (E) HeLa FUCCI cells infected with C. trachomatis were treated with OA and ATMi and pulsed with BrdU for 1 h before immunofluorescence labeling with BrdU antibody was performed. DNA was counterstained with Hoechst stain. Images were acquired with an automated microscope. Bar graph data represent percentages of cells in G_2_/M phase. Cells treated with 50 µM etoposide for the last 2 h served as a positive control. Graphs represent means + SEM. ****, *P* < 0.0001 (determined by Student's *t* test). All data are representative of results from three independent experiments. Densitometry values for pChk2 immunoblots were normalized to the β-actin results, and the relative fold change values compared to the uninfected and untreated control are shown.

ATM acts as a dual-function protein in DNA damage repair as well as in transduction of signals eliciting cell cycle checkpoints to provide enough time for DNA repair and/or apoptosis induction, depending on the extent of DNA damage (8, 15, 23). Here we assessed whether activation of ATM following PP2A inhibition restores the functional cell cycle checkpoint in C. trachomatis*-*infected cells. To this end, cells infected for 45 h with or without additional treatment of OA during the last 6, 12, 20, or 24 h were subjected to immunoblot analysis for phosphorylation of the checkpoint protein Chk2, an effector of active ATM kinase. Levels of phosphorylated Chk2 increased in infected cells treated with OA in contrast to untreated infected cells, depending on the duration of treatment (Fig. 3A). Further, we analyzed whether activation of Chk2 protein would culminate in cell cycle arrest. Since C. trachomatis is an intracellular pathogen, cell cycle analysis based on DNA content using a fluorescence-activated cell sorting (FACS) approach remains suboptimal, as C. trachomatis DNA interferes with the quantification. For this reason, we used the novel and powerful FUCCI (fluorescence ubiquitin cell cycle indicator) cell system, which utilizes fluorescent proteins in combination with two components of the DNA replication control system of higher eukaryotes: the licensing factor Cdt1 and its inhibitor geminin. The levels of abundance of Cdt1 and geminin show inverse patterns during the cell cycle, with opposing effects on DNA replication. Cdt1 protein peaks in G_1_ phase just before the onset of DNA replication and declines abruptly after S-phase initiation. In contrast, geminin levels are high during S and G_2_ phase but are low during late mitosis and G_1_ phase (24). These HeLa FUCCI cells (24), which exhibit green fluorescence during S/G_2_/M phase and red fluorescence during G_1_ phase, in combination with additional immunostaining for S phase using bromodeoxyuridine (BrdU) antibody, enable the identification of cells that are in G_1_, S, or G_2_/M phase. Using automated image acquisition and analysis of FUCCI HeLa cells additionally labeled with BrdU and Hoechst, we found that cells infected with C. trachomatis proliferated without cell cycle arrest (Fig. 3B and C). However, activation of ATM via OA treatment in infected cells led to increased accumulation of cells in G_2_/M phase. Representative images of C. trachomatis-infected HeLa FUCCI cells with or without OA (Fig. 3B) show increased levels of green fluorescent protein (GFP)-positive cells that occurred in an OA concentration-dependent manner (Fig. 3B). Further, quantification of GFP-positive and BrdU-negative cells indicated G_2_/M cell cycle arrest in response to PP2A inhibition (Fig. 3C). Cells treated with etoposide served as a positive control for DSB-induced G_2_/M cell cycle arrest. Further, we analyzed if the observed cell cycle checkpoint activation and enhanced G_2_/M arrest are dependent on ATM activation. To this end, C. trachomatis*-*infected cells were treated with OA alone or in combination with the ATM kinase inhibitor KU-55933 (ATMi) and analyzed for pChk2 by immunoblotting and for cell cycle profile using HeLa FUCCI cells. Increases in pChk2 levels, as well as increased proportions of cells in G_2_/M phase in infected cells treated with OA, are prevented when ATM activation is inhibited by ATMi (Fig. 3D and E). Thus, inhibition of PP2A rescues the C. trachomatis-imposed suppression of ATM activation*-*mediated cell cycle arrest.

### PP2A impairs DNA repair by homologous recombination in *Chlamydia*-infected cells.

**FIG 4:**
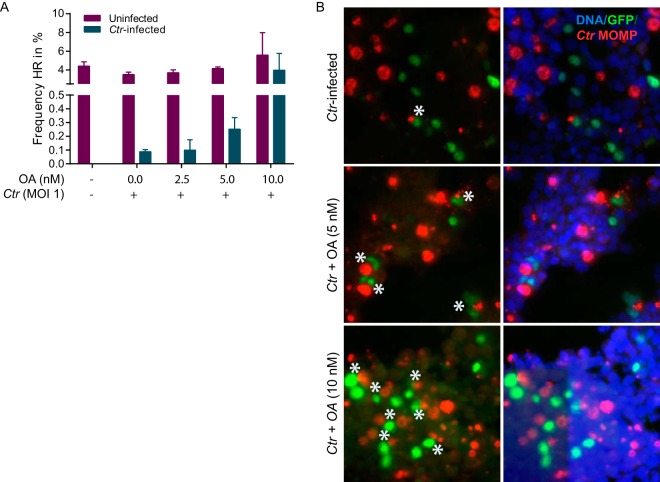
C. trachomatis-induced DSBs fail to activate homologous recombination, while inhibition of PP2A restores the HR pathway. (A and B) HEK293 homologous recombination (HR) repair GFP reporter (DR-GFP) cells were infected with C. trachomatis and treated with OA at 2 h p.i. followed by immunofluorescence labeling for MOMP at 40 h p.i. DNA was counterstained with Hoechst stain. (A) Numbers of infected GFP^+^ cells as determined using an automated Scan R microscope. (B) Representative images of labeled cells. Asterisks indicate HR-positive infected cells. Data are representative of results from three independent experiments. Graph data represent means + SEM.

**FIG 5:**
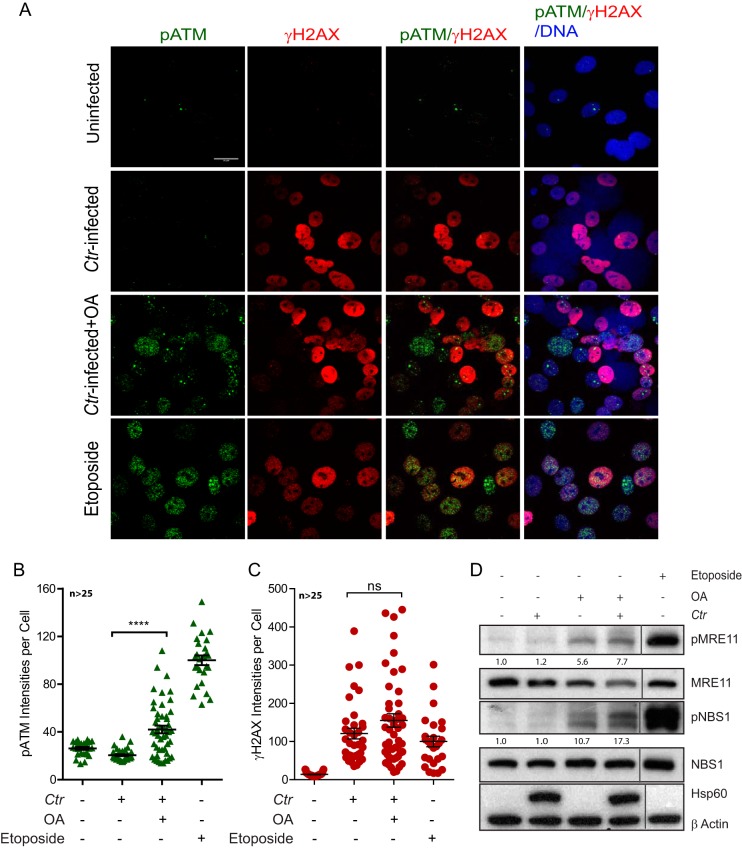
Upon PP2A inhibition, activated ATM recruits partially to DSBs and phosphorylates its substrates in C. trachomatis-infected cells. (A to C) End1 E6/E7 cells were infected with C. trachomatis for 36 h and treated with OA for the last 20 h, followed by immunofluorescence analysis. (A) Representative confocal images of γH2AX (red), pATM (green), and DNA (blue). Bar, 20 μm. (B and C) Mean pixel intensities of γH2AX and pATM per cell quantified using ImageJ. Data represent means ± SEM for (B) pATM (green) and (C) γH2AX (red) for at least 25 cells per condition. Only γH2AX-positive cells were quantified under the C. trachomatis-infected condition. (D) End1 E6/E7 cells infected with C. trachomatis for 45 h and treated with OA for the last 2 h were analyzed by immunoblotting for pMRE11 (S676), MRE11, pNBS1 (S343), NBS1, chlamydial Hsp60, and β-actin. Data are representative of results from three independent experiments; Densitometry values for pMRE11 and pNBS1 immunoblots were normalized to the β-actin values, and data representing the relative fold change compared to an uninfected and untreated control are shown. ****, *P* < 0.0001; ns, *P* > 0.05 (analyzed by Student's *t* test).

To investigate the efficiency of HR repair in the face of C. trachomatis-induced DSBs, we used the HEK293 DR-GFP reporter cell line. This cell line has an I-SceI site integrated into a full-length GFP gene (SceGFP), which allows the introduction of a single DSB via the rare-cutting endonuclease I-SceI, thus disrupting the GFP gene (25). Repair of this DSB by HR, using a downstream internal truncated GFP fragment as the template, results in a functional GFP gene. Using the HEK293 DR-GFP cell line, we further developed an automated microscopic assay using the ScanR system (Olympus) for simultaneous visualization and scoring of GFP-positive infected and uninfected cells from the same microscopic image. HEK293 DR-GFP cells were infected with C. trachomatis, and the numbers of GFP-positive cells were compared to the levels measured in uninfected cells. Interestingly, the numbers of GFP-positive cells in infected and uninfected cultures remained the same. However, even in the C. trachomatis-infected cultures, a GFP signal was observed only in cells that had remained noninfected, indicating an almost complete abrogation of HR in infected cells (Fig. 4). PP2A inhibition led to recovery of HR in infected cells in a concentration-dependent manner (Fig. 4A and 5B).

Further, the restored functional HR is associated with the increased recruitment of activated ATM to the DSBs after PP2A inhibition in C. trachomatis-infected cells. Immunofluorescence analysis of pATM, γH2AX, and DNA in cells infected for 36 h with or without addition of OA during the last 20 h, or treated with etoposide for 2 h as a positive control, showed that, in contrast to etoposide-treated cells, C. trachomatis*-*infected cells lacked pATM foci at DSB sites (Fig. 5A). OA-treated cells showed increased localization of pATM to the damaged DNA after infection (Fig. 5A to C).

Proper HR repair depends on ATM-mediated phosphorylation of MRE11 S676 and S678, which is required for end resection by exonuclease 1, as well as S343 on MRN complex member NBS1 (16, 26, 27). Therefore, we used immunoblotting to analyze phosphorylation of MRE11 at S676 (pMRE11) and NBS1 at S343 (pNBS1) in C. trachomatis-infected cells with or without OA treatment. No increase in pMRE11 and pNBS1 levels could be observed compared to uninfected control cells. In contrast, C. trachomatis-infected cells treated with OA showed enhanced levels of pMRE11 and pNBS1 (Fig. 5D), in congruence with the increase in pATM levels described above (Fig. 1C and D and 5A). Taking the data together, our results show that C. trachomatis infection suppresses the HR repair response to DSBs as a consequence of PP2A-mediated inactivation of ATM signaling, thereby leading to a predisposition to host cell mutagenesis.

## DISCUSSION

This report reveals that *Chlamydia*, despite inducing DSBs, modulates a host regulatory inhibition mechanism to alter the usual DDR and homologous recombination repair processes to ensure its survival, while potentiating the threat to the genome integrity of its host cells.

DNA damage induces a series of phosphorylation events, mainly of serine (Ser) and threonine (Thr) residues, leading to the activation of checkpoint proteins and DNA repair factors (18). Phosphorylation of ATM on Ser1981 following DSB induction leads to its activation as an apical activator and mobilizer of DDR proteins (15, 23). Failure of ATM activation can have severe effects on genomic stability. We have previously reported that C. trachomatis induces enhanced ROS production, which is essential for its development but as a consequence induces DSBs in the host DNA that fail to activate either ATM or MRE11 (8). C. trachomatis is also known to modulate mitogen-activated protein kinase (MAPK) signaling, contributing to aberrant cell proliferation despite the presence of DSBs (4, 8).

Several studies have proposed alternative mechanisms for ATM activation, including direct interaction with single-stranded DNA or oligonucleotides at DSBs (16). However, these mechanisms clearly fail to elicit an ATM response in C. trachomatis-infected cells despite the presence of DSBs. While the role of kinases in the DDR has been well established, the complex roles of protein dephosphorylation by phosphatases are only just emerging. Dephosphorylation events have been implicated in keeping DDR factors inactive during normal cell growth (28, 29) and in the inactivation of checkpoint arrest following DNA repair. Further, spatial and temporal regulation of dephosphorylation events mediated by phosphatases is crucial for the DDR (28).

Activation of ATM in response to irradiation-induced DSBs has been shown to require disruption of its constitutive association with PP2A (19). In contrast, our results show that C. trachomatis interferes with the normal PP2A response to DSBs, thus inhibiting the ATM-mediated DDR. ATM activity is further regulated by protein phosphatases PP1 and PP5, with PP1 inhibiting ATM activity (19, 30), while PP5 activates it by an as-yet-unknown mechanism (31). However, we demonstrated that PP2A is specifically involved in suppressing ATM activation in C. trachomatis-infected cells, since siRNA-mediated knockdown of the catalytic alpha subunit of PP2A led to activation of ATM. Nevertheless, continued PP2A activity in C. trachomatis-infected cells might have additional effects on host cells, as it is known to play important roles in regulating multiple signaling pathways such as the phosphatidylinositol 3-kinase (PI3K), Wnt, and RAS pathways (32).

We investigated in more depth the mechanism by which C. trachomatis infection regulates PP2A. PP2A activity is regulated via posttranslational modifications of its catalytic subunit. Methylation of the catalytic subunit enhances activity by facilitating binding of the regulatory B subunit, an essential event for interaction with target proteins (20). In contrast, phosphorylation of the catalytic subunit PP2A-C at pY307 inhibits binding of the B subunit and interaction with the target protein. The phosphatase inhibitor OA enhances phosphorylation at PP2A-C pY307, potently inactivating PP2A (33). Here, we found that while C. trachomatis*-*infected cells show minimal phosphorylation of PP2A-C Y307, comparably to uninfected cells, OA treatment leads to inactivation of PP2A by increasing PP2A-C pY307 and dissociation of ATM, resulting in ATM phosphorylation.

Phosphorylated ATM modulates the activity of several downstream effector protein kinases, leading to the coordinated activation of the DNA repair machinery and cell cycle checkpoints (34). Checkpoint kinase 2 (Chk2) is a well-known effector of pATM (35). ATM activation following irradiation-induced DNA damage leads to Chk2 phosphorylation and G_2_/M cell cycle arrest (35, 36). In C. trachomatis-infected cells, PP2A-mediated suppression of ATM phosphorylation prevents Chk2 phosphorylation and cell cycle arrest. Since HR occurs only during the S and G_2_ phases, when sister chromatids are available as templates, lack of ATM and the consequent checkpoint activation suppress HR and shift DSB repair to the error-prone non-homologous end joining (NHEJ) pathway, which repairs breaks during all phases of the cell cycle as it does not require sequence homology (14, 37, 38).

Once ATM is phosphorylated, it undergoes spatial relocalization to the nucleus, where it initiates repair by phosphorylating proteins that are recruited to DSBs. These form discrete foci of phosphorylated ATM substrates (such as histone H2AX, NBS1, and MRE11) (15). This recruitment of pATM is suppressed by C. trachomatis infection, but our findings show that it can be partially rescued by inactivating PP2A. As a consequence, ATM-mediated phosphorylation of MRE11 and NBS1 is also restored, allowing HR repair of DSBs to proceed. We have shown previously that the ATM substrate H2AX is phosphorylated by the NHEJ pathway effector DNA-dependent protein kinase catalytic subunit (DNA-PKcs) in response to C. trachomatis*-*induced DSBs in the absence of ATM activation (8). This suggests that the NHEJ repair pathway in C. trachomatis-infected cells is predominantly active. In congruence, infected cells exhibit defective repair of DSBs, leading to loss of nucleotides and mismatch incorporation of nucleotides (8). Furthermore, *Chlamydia* infection causes cytokinesis defects, leading to multinucleated host cells, increased supernumerary centrosomes, abnormal spindle poles, and chromosomal segregation defects (39, 40) that could culminate in genomic instability. In the present study, we directly confirmed—using a reporter cell line—that HR does not take place in C. trachomatis-infected cells, as ATM activation is blocked by PP2A.

Thus, to promote cell cycle progression and survival for its propagation, C. trachomatis interferes with the usual function of PP2A, thereby suppressing the ATM-HR axis responsible for high-fidelity repair of DSBs. Further, the functions of PP2A and ATM are known not only to regulate the cellular response to DSBs but also to function in various branches of metabolism and cell signaling (41), indicating the magnitude of the effect on diverse signaling cascades in C. trachomatis-infected cells—thus leading to a predisposition to mutagenesis and loss of cellular homeostasis.

## MATERIALS AND METHODS

### Cell culture.

End1 E6/E7 (End1) (ATCC CRL-2615), FUCCI HeLa cells, and HEK293 DR reporter cells were cultured in HEPES-buffered Dulbecco’s modified Eagle’s medium (DMEM) (Gibco) supplemented with 10% fetal calf serum (FCS) (Biochrome), 2 mM glutamine, and 1 mM sodium pyruvate, at 37°C in a humidified incubator containing 5% CO_2_.

### *Chlamydia* infections.

C. trachomatis L2 (ATCC VR-902B) stocks were prepared as described earlier (4). Unless otherwise stated, *Chlamydia* infection experiments were performed at a multiplicity of infection (MOI) of 5 in infection medium (DMEM supplemented with 5% FCS, 2 mM glutamine, and 1 mM sodium pyruvate). The medium was exchanged at 2 h postinfection (p.i.), and cells were grown at 35°C in 5% CO_2_.

### SDS-PAGE and Western blotting.

Cells grown in six-well plates and treated per experimental requirements were washed with phosphate-buffered saline (PBS) and lysed with 300 μl of SDS sample buffer (3% 2-mercaptoethanol, 20% glycerin, 0.05% bromophenol blue, 3% SDS). Cell lysates were collected and incubated at 95°C for 10 min. Samples were stored at −20°C until required. SDS-PAGE and Western blotting were performed as described earlier (4).

### Indirect immunofluorescence microscopy.

Indirect immunofluorescence microscopy was performed as described in reference 8. Briefly, cells incubated in CSK buffer {10 mM PIPES [piperazine-*N*,*N*′-bis(2-ethanesulfonic acid)] (pH 6.8), 100 mM NaCl, 300 mM sucrose, 3 mM MgCl_2_, 1 mM EGTA, 0.5% TritonX-100} on ice for 10 min and then fixed with 2% paraformaldehyde (PFA) at room temperature for 30 min. Cells were washed with PBST (PBS–0.1% Tween 20) and then incubated in blocking buffer (3% bovine serum albumin [BSA]–PBST) for 30 min followed by 1 h of incubation at room temperature with primary antibodies. Cells were washed and incubated for 1 h at room temperature with the appropriate fluorochrome-conjugated secondary antibodies. Cells were also stained for DNA with Draq5-PBS or Hoechst stain-PBS for 5 min before being mounted with Mowiol. The fluorochromes were visualized with Cy2, Cy3, and Cy5 filters. A series of images with z-stacks were acquired with a laser scanning confocal microscope (Leica) and further processed with Image J and Photoshop CS3 (Adobe Systems). Integrated intensities were calculated using ImageJ software.

### Proximity ligation assay.

Proximity ligation assays were performed as previously described (4). Briefly, End1E67E7 cells grown on coverslips in 24-well plates were infected with C. trachomatis. Cells were washed twice with PBS and then fixed with ice-cold methanol overnight at 4°C. Incubation with antibodies against ATM and PP2A-A was performed with a Duolink In Situ Orange goat/rabbit starter kit (Sigma) according to the manufacturer’s instructions. A series of images with z-stacks were acquired with a laser scanning confocal microscope (Leica), analyzed with ImageJ software, and further processed by Photoshop CS3 (Adobe Systems).

### Cell cycle analysis using FUCCI HeLa cells.

Fucci HeLa cells were seeded in 96-well plates (5,000 cells/well) and then infected with C. trachomatis L2 on the following day. Cells were then treated with OA with or without 10 µM ATM inhibitor (KU-55933). Cells were treated with BrdU at a 1:1,000 dilution in 5% FCS–DMEM for 1 h before PFA fixation was performed followed by incubation with 2 M HCL for 20 min and then 0.1 M sodium borate (Na_2_B_4_O_7_) (pH 8.5) for 2 min. The cells were permeabilized and stained with anti-BrdU (1:1,000) primary antibody followed by fluorochrome-conjugated secondary antibody and Hoechst stain. Finally, images were acquired by the use of an automated microscope and Cy2, Cy3, Cy5, and DAPI (4′,6-diamidino-2-phenylindole) filters, and data were analyzed by the use of ScanR software and a custom-developed image analysis assay.

### Antibodies and chemicals.

The antibodies and sources used were as follows: anti-BrdU from GE Healthcare; pChk2 (Th68), ATM (D2E2 pATM) (Ser1981), and pMre11 (Ser676) from Cell Signaling; gH2AX (Ser139) from Upstate; pATM (Ser1981), Mre11, Chk1, NBS1, pNBS1 (S343), and PP2A C (p-Y307) from Abcam; goat-anti-*Chlamydia* major outer membrane protein (MOMP) from AbD Serotec; *Chlamydia* HSP60 from Enzo Life Sciences; β-actin from Sigma; and mouse anti-*Chlamydia* MOMP KK12 from the University of Washington. Secondary antibodies conjugated to horseradish peroxidase (HRP) were purchased from Amersham Biosciences, and secondary antibodies labeled with Cy2, Cy3, or Cy5 were from Jackson Immuno Research Laboratories. Hoechst stain was purchased from Sigma and Draq5 from Cell Signaling. All reagents were used for Western blotting or immunofluorescence at the dilutions recommended by the manufacturers. Chemicals were obtained from the following sources: okadaic acid (OA) from Santa Cruz, etoposide and FTY720 from Sigma, and ATM kinase KU-55933 inhibitor from Merck Millipore.

### Neutral comet assay.

Single-cell comet assays were performed according to the instructions of the manufacturer (Trevigen). Briefly, cells were resuspended in cold PBS mixed with low-melting agarose at a ratio of 1:10, and 50 µl of cell suspension was spread on a comet slide. Slides were placed in lysis buffer followed by electrophoresis, transferred to 70% ethanol, and stained with SYBR green. Nuclei were visualized using epifluorescent illumination on a Zeiss microscope. The DNA damage was quantified by determining the percentage of DNA in the tail using Comet Score (TriTek) software. Graphs were generated using GraphPad Prism 5 (GraphPad Software, Inc.).

### siRNA transfection and knockdown analysis.

siRNAs used in this study were purchased from Qiagen. siRNA transfections were performed as described previously (4) to reach a final siRNA concentration of 20 nM, using Hiperfect transfection reagent according to the manufacturer’s guidelines. Two days posttransfection, the cells were used for different experiments or to determine knockdown efficiency by reverse transcription-quantitative PCR (RT-qPCR).

### HR repair analysis in HEK293 (DR-GFP) cell line.

HEK293 (DR-GFP) cells (2 × 10^4^) were seeded in 96-well plates coated with a 1:100 dilution of collagen. The following day, medium was aspirated and 100 µl fresh antibiotic-free medium added 2 h before transfection. Cells were transfected with I-SceI expression plasmid by diluting 0.25 µg of vector and 0.5 µl of Lipofectamine 2000 in 50 µl Optimem medium, followed by 25 min of incubation of the transfection mixture at room temperature. Then, 50 µl of transfection complexes per well was added to the 96-well plate and incubated at 37°C for 3 h. The cells were washed with medium and infected with C. trachomatis *L2* at the indicated MOI. After 2 h of infection, cells were treated with 2.5 nM, 5 nM, or 10 nM OA. Finally, after 40 h of infection, cells were fixed with 3.7**%** PFA and permeabilized with 1% FCS–0.05% Tween 20–2% Triton X-100–1× PBS for 2 days at 4°C. The cells were then stained with goat C. trachomatis MOMP antibody followed by fluorochrome-conjugated secondary antibody and Hoechst stain for nuclear staining. Images were acquired with an automated microscope and analyzed by the use of ScanR software using a custom-developed image analysis assay.

### Statistical analysis.

Statistical analyses were performed using GraphPad Prism 5 software. Student's *t* test was used to determine *P* values. Significance levels are indicated throughout with asterisks (****, *P* < 0.0001; ***, *P* < 0.001; **, *P* < 0.01; *, *P* < 0.05; ns, *P* > 0.05 [not significant]).

## References

[1] Arnheim Dahlstrom L, Andersson K, Luostarinen T, Thoresen S, Ogmundsdottir H, Tryggvadottir L, Wiklund F, Skare GB, Eklund C, Sjolin K, Jellum E, Koskela P, Wadell G, Lehtinen M, Dillner J 2011 Prospective seroepidemiologic study of human papillomavirus and other risk factors in cervical cancer. Cancer Epidemiol Biomarkers Prev 20:2541–2550. doi:10.1158/1055-9965.EPI-11-0761. 21994401

[2] Koskela P, Anttila T, Bjorge T, Brunsvig A, Dillner J, Hakama M, Hakulinen T, Jellum E, Lehtinen M, Lenner P, Luostarinen T, Pukkala E, Saikku P, Thoresen S, Youngman L, Paavonen J 2000 Chlamydia trachomatis infection as a risk factor for invasive cervical cancer. Int J Cancer 85:35–39. doi:10.1002/(SICI)1097-0215(20000101)85:1<35::AID-IJC6>3.0.CO;2-A. 10585579

[3] Shanmughapriya S, Senthilkumar G, Vinodhini K, Das BC, Vasanthi N, Natarajaseenivasan K 2012 Viral and bacterial aetiologies of epithelial ovarian cancer. Eur J Clin Microbiol Infect Dis 31:2311–2317. doi:10.1007/s10096-012-1570-5. 22402815

[4] Gurumurthy RK, Maurer AP, Machuy N, Hess S, Pleissner KP, Schuchhardt J, Rudel T, Meyer TF 2010 A loss-of-function screen reveals Ras- and Raf-independent MEK-ERK signaling during Chlamydia trachomatis infection. Sci Signal 3:ra21. doi:10.1126/scisignal.2000651. 20234004

[5] Gonzalez E, Rother M, Kerr MC, Al-Zeer MA, Abu-Lubad M, Kessler M, Brinkmann V, Loewer A, Meyer TF 2014 Chlamydia infection depends on a functional MDM2-p53 axis. Nat Commun 5:5201. doi:10.1038/ncomms6201. 25392082 PMC4243245

[6] Siegl C, Prusty BK, Karunakaran K, Wischhusen J, Rudel T 2014 Tumor suppressor p53 alters host cell metabolism to limit Chlamydia trachomatis infection. Cell Rep 9:918–929. doi:10.1016/j.celrep.2014.10.004. 25437549

[7] Padberg I, Janßen S, Meyer TF 2013 Chlamydia trachomatis inhibits telomeric DNA damage signaling via transient hTERT upregulation. Int J Med Microbiol 303:463–474. doi:10.1016/j.ijmm.2013.06.001. 23830072

[8] Chumduri C, Gurumurthy RK, Zadora PK, Mi Y, Meyer TF 2013 Chlamydia infection promotes host DNA damage and proliferation but impairs the DNA damage response. Cell Host Microbe 13:746–758. doi:10.1016/j.chom.2013.05.010. 23768498

[9] Gurumurthy RK, Chumduri C, Karlas A, Kimmig S, Gonzalez E, Machuy N, Rudel T, Meyer TF 2014 Dynamin-mediated lipid acquisition is essential for Chlamydia trachomatis development. Mol Microbiol 94:186–201. doi:10.1111/mmi.12751. 25116793

[10] Chumduri C, Gurumurthy RK, Zietlow R, Meyer TF 2016 Subversion of host genome integrity by bacterial pathogens. Nat Rev Mol Cell Biol 17:659–673. doi:10.1038/nrm.2016.100. 27534801

[11] Mpiga P, Ravaoarinoro M 2006 Chlamydia trachomatis persistence: an update. Microbiol Res 161:9–19. doi:10.1016/j.micres.2005.04.004. 16338585

[12] Jackson SP, Bartek J 2009 The DNA-damage response in human biology and disease. Nature 461:1071–1078. doi:10.1038/nature08467. 19847258 PMC2906700

[13] O'Driscoll M, Jeggo PA 2006 The role of double-strand break repair - insights from human genetics. Nat Rev Genet 7:45–54. doi:10.1038/nrg1746. 16369571

[14] Bakr A, Oing C, Kocher S, Borgmann K, Dornreiter I, Petersen C, Dikomey E, Mansour WY 2015 Involvement of ATM in homologous recombination after end resection and RAD51 nucleofilament formation. Nucleic Acids Res 43:3154–3166. doi:10.1093/nar/gkv160. 25753674 PMC4381069

[15] Shiloh Y, Ziv Y 2013 The ATM protein kinase: regulating the cellular response to genotoxic stress, and more. Nat Rev Mol Cell Biol 14:197–210. doi:10.1038/nrm3546. 23847781

[16] Kijas AW, Lim YC, Bolderson E, Cerosaletti K, Gatei M, Jakob B, Tobias F, Taucher-Scholz G, Gueven N, Oakley G, Concannon P, Wolvetang E, Khanna KK, Wiesmüller L, Lavin MF 2015 ATM-dependent phosphorylation of MRE11 controls extent of resection during homology directed repair by signalling through exonuclease 1. Nucleic Acids Res 43:8352–8367. doi:10.1093/nar/gkv754. 26240375 PMC4787824

[17] Sharma M, Rudel T 2009 Apoptosis resistance in Chlamydia-infected cells: a fate worse than death? FEMS Immunol Med Microbiol 55:154–161. doi:10.1111/j.1574-695X.2008.00515.x. 19281566

[18] Bennetzen MV, Larsen DH, Bunkenborg J, Bartek J, Lukas J, Andersen JS 2010 Site-specific phosphorylation dynamics of the nuclear proteome during the DNA damage response. Mol Cell Proteomics 9:1314–1323. doi:10.1074/mcp.M900616-MCP200. 20164059 PMC2877989

[19] Goodarzi AA, Jonnalagadda JC, Douglas P, Young D, Ye R, Moorhead GB, Lees-Miller SP, Khanna KK 2004 Autophosphorylation of ataxia-telangiectasia mutated is regulated by protein phosphatase 2A. EMBO J 23:4451–4461. doi:10.1038/sj.emboj.7600455. 15510216 PMC526470

[20] Longin S, Zwaenepoel K, Louis JV, Dilworth S, Goris J, Janssens V 2007 Selection of protein phosphatase 2A regulatory subunits is mediated by the C terminus of the catalytic subunit. J Biol Chem 282:26971–26980. doi:10.1074/jbc.M704059200. 17635907

[21] Seshacharyulu P, Pandey P, Datta K, Batra SK 2013 Phosphatase: PP2A structural importance, regulation and its aberrant expression in cancer. Cancer Lett 335:9–18. doi:10.1016/j.canlet.2013.02.036. 23454242 PMC3665613

[22] Guo CY, Brautigan DL, Larner JM 2002 ATM-dependent dissociation of B55 regulatory subunit from nuclear PP2A in response to ionizing radiation. J Biol Chem 277:4839–4844. doi:10.1074/jbc.M110092200. 11723136

[23] Bernstein C, Bernstein H, Payne CM, Garewal H 2002 DNA repair/pro-apoptotic dual-role proteins in five major DNA repair pathways: fail-safe protection against carcinogenesis. Mutat Res 511:145–178. doi:10.1016/S1383-5742(02)00009-1. 12052432

[24] Sakaue-Sawano A, Kurokawa H, Morimura T, Hanyu A, Hama H, Osawa H, Kashiwagi S, Fukami K, Miyata T, Miyoshi H, Imamura T, Ogawa M, Masai H, Miyawaki A 2008 Visualizing spatiotemporal dynamics of multicellular cell-cycle progression. Cell 132:487–498. doi:10.1016/j.cell.2007.12.033. 18267078

[25] Gunn A, Stark JM 2012 I-SceI-based assays to examine distinct repair outcomes of mammalian chromosomal double strand breaks. Methods Mol Biol 920:379–391. doi:10.1007/978-1-61779-998-3_27. 22941618

[26] Zhao S, Weng YC, Yuan SS, Lin YT, Hsu HC, Lin SC, Gerbino E, Song MH, Zdzienicka MZ, Gatti RA, Shay JW, Ziv Y, Shiloh Y, Lee EY 2000 Functional link between ataxia-telangiectasia and Nijmegen breakage syndrome gene products. Nature 405:473–477. doi:10.1038/35013083. 10839544

[27] Tauchi H, Kobayashi J, Morishima K, van Gent DC, Shiraishi T, Verkaik NS, vanHeems D, Ito E, Nakamura A, Sonoda E, Takata M, Takeda S, Matsuura S, Komatsu K 2002 Nbs1 is essential for DNA repair by homologous recombination in higher vertebrate cells. Nature 420:93–98. doi:10.1038/nature01125. 12422221

[28] Peng A, Maller JL 2010 Serine/threonine phosphatases in the DNA damage response and cancer. Oncogene 29:5977–5988. doi:10.1038/onc.2010.371. 20838380

[29] Lee DH, Chowdhury D 2011 What goes on must come off: phosphatases gate-crash the DNA damage response. Trends Biochem Sci 36:569–577. doi:10.1016/j.tibs.2011.08.007. 21930385 PMC3402068

[30] Peng A, Lewellyn AL, Schiemann WP, Maller JL 2010 Repo-man controls a protein phosphatase 1-dependent threshold for DNA damage checkpoint activation. Curr Biol 20:387–396. doi:10.1016/j.cub.2010.01.020. 20188555 PMC2860455

[31] Ali A, Zhang J, Bao S, Liu I, Otterness D, Dean NM, Abraham RT, Wang XF 2004 Requirement of protein phosphatase 5 in DNA-damage-induced ATM activation. Genes Dev 18:249–254. doi:10.1101/gad.1176004. 14871926 PMC338278

[32] Wlodarchak N, Xing Y 2016 PP2A as a master regulator of the cell cycle. Crit Rev Biochem Mol Biol 51:162–184. doi:10.3109/10409238.2016.1143913. 26906453 PMC4905575

[33] Chen J, Martin BL, Brautigan DL 1992 Regulation of protein serine-threonine phosphatase type-2A by tyrosine phosphorylation. Science 257:1261–1264. doi:10.1126/science.1325671. 1325671

[34] Ciccia A, Elledge SJ 2010 The DNA damage response: making it safe to play with knives. Mol Cell 40:179–204. doi:10.1016/j.molcel.2010.09.019. 20965415 PMC2988877

[35] Matsuoka S, Huang M, Elledge SJ 1998 Linkage of ATM to cell cycle regulation by the Chk2 protein kinase. Science 282:1893–1897. doi:10.1126/science.282.5395.1893. 9836640

[36] Tatsuka M, Nikaido O, Tatsumi K, Takebe H 1989 X-ray-induced G_2_ arrest in ataxia telangiectasia lymphoblastoid cells. Mutat Res 214:321–328. 2797028 10.1016/0027-5107(89)90174-7

[37] Bergs JW, Krawczyk PM, Borovski T, ten Cate R, Rodermond HM, Stap J, Medema JP, Haveman J, Essers J, van Bree C, Stalpers LJ, Kanaar R, Aten JA, Franken NA 2013 Inhibition of homologous recombination by hyperthermia shunts early double strand break repair to non-homologous end-joining. DNA Repair (Amst) 12:38–45. doi:10.1016/j.dnarep.2012.10.008. 23237939

[38] Ira G, Pellicioli A, Balijja A, Wang X, Fiorani S, Carotenuto W, Liberi G, Bressan D, Wan L, Hollingsworth NM, Haber JE, Foiani M 2004 DNA end resection, homologous recombination and DNA damage checkpoint activation require CDK1. Nature 431:1011–1017. doi:10.1038/nature02964. 15496928 PMC4493751

[39] Brown HM, Knowlton AE, Grieshaber SS 2012 Chlamydial infection induces host cytokinesis failure at abscission. Cell Microbiol 14:1554–1567. doi:10.1111/j.1462-5822.2012.01820.x. 22646503 PMC3443326

[40] Grieshaber SS, Grieshaber NA, Miller N, Hackstadt T 2006 Chlamydia trachomatis causes centrosomal defects resulting in chromosomal segregation abnormalities. Traffic 7:940–949. doi:10.1111/j.1600-0854.2006.00439.x. 16882039

[41] Ferrari E, Bruhn C, Peretti M, Cassani C, Carotenuto WV, Elgendy M, Shubassi G, Lucca C, Bermejo R, Varasi M, Minucci S, Longhese MP, Foiani M 2017 PP2A controls genome integrity by integrating nutrient-sensing and metabolic pathways with the DNA damage response. Mol Cell 67:266–281.e4. doi:10.1016/j.molcel.2017.05.027. 28648781 PMC5526790

